# Review of Sharp Recanalization Techniques in Central Venous Occlusions

**DOI:** 10.1007/s00270-024-03789-8

**Published:** 2024-07-30

**Authors:** Tony Rizk, Antony Gayed, Stephen Stringfellow, Yara Younan, Ricardo Yamada, Marcelo Guimaraes

**Affiliations:** Division of Vascular and Interventional Radiology, Medical University of South Carolina, Charleston, SC USA

**Keywords:** Central venous occlusion, Sharp recanalization, Outcomes

## Abstract

Benign central venous occlusions are frequently associated with long-term central venous access. Most of these occlusions can be recanalized with conventional endovascular technique. When conventional technique fails, sharp recanalization techniques (SRTs) can increase technical success. The SRTs include single low-profile needles, needle coaxial systems, re-entry catheter, the back end of stiff guidewires, and systems that can deliver radiofrequency energy or laser. This review on SRTs presents technical details and outcomes of the most common techniques used in central venous recanalization.

## Introduction

Benign central venous occlusions (CVO) are mostly catheters-induced and have an incidence between 3 and 38% [1, 2]. Arm swelling, facial swelling, and dyspnea are among the most common complaints, secondary to venous hypertension. Chronic and symptomatic CVOs are often successfully treated with conventional endovascular techniques, but limited literature on failure rates suggest that to be between 11 and 24% of patients [3, 4]. Alternatives to failed conventional techniques include observation, surgical fistula or graft ligation, surgical venous-venous bypass, and advanced endovascular recanalization such as sharp recanalization techniques (SRTs). SRT was first described by Ferral in 1996 [5]. The most common crossing tools used in SRT include the back end of stiff guidewire, needle-assisted techniques (Chiba, Colapinto, Transjugular intrahepatic portosystemic shunt (TIPS) set, Transseptal and coaxial systems), re-entry tool such as the Outback device (Cordis, Miami Lakes, FL/USA), and the use of radiofrequency (RF) or laser (Table 1). The success rates of SRTs range from 83 to 100%, and major complication rates range from 0 to 29% [6]. The most common SRTs and outcomes are reviewed here.

**Table 1:** Summary of the sharp recanalization techniques and outcomes

Sharp recanalization technique	Approach (cranial–caudal versus caudal–cranial)	Ideal indication	Advantage(s)	Disadvantage(s)	Success rate	Major complication rate
Back end of stiff guidewire [10–13]	Any	Short, straight trajectories	Multiple access points, low cost, availability	Limited steerability, may require substantial crossing force	74.1–100%	0.5–1%
Chiba needle [10, 13, 16, 17]	Cranial–caudal (IJ only)	Short, straight trajectories	Low cost, availability	Limited steerability, too short for upper arm or groin access, may require substantial crossing force	85.7–100%	5.1–7.1%
Colapinto needle, TIPS set [20–22, 24]	Cranial–caudal (IJ or brachial)	Short, straight trajectories	Less crossing force relative to Chiba needle, manually curved	Limited opportunity for groin approach if curved, large diameter, increased procedural expenses	93.9–100%	14.3%
Transseptal needle [11, 25, 26]	Any	Curved short trajectories, straight long trajectories	Multiple access points, steerability, small diameter	May require substantial crossing force	77.1–100%	2.4–28.5%
Coaxial needle system [6]	Cranial–caudal (IJ only)	Short, straight trajectories in right BCV and SVC occlusions	Low cost, availability accommodates 0.018 in. guidewire system, steerability	Too short for upper arm or groin access, may require substantial crossing force	94%	3%
Outback re-entry device [27, 28]	Any	Curved occlusions	Multiple access points, steerability	Increased procedural expenses, limited data on this off-label use	90.9–100%	0%
RF Wire [8, 30–33]	Any	Straight and curved occlusions	Multiple access points, steerability, easily traverses fibrotic occlusions	Increased procedural expenses, limited availability	80–100%	2.4–8.3%
Excimer laser [34]	Cranial–caudal (ipsilateral to AVF)	Fibrotic occlusions	Debulking allows full stent expansion	Increased procedural expenses, limited availability, limited date on this off-label use	N/A	N/A

## Universal Principles of Sharp Recanalization Techniques

There are common basic principles across most SRT. Dual venous accesses are obtained from the upper [Internal Jugular (IJ), Subclavian, upper arm veins, arterio-venous fistula (AVF) or graft (AVG) access] and lower parts of the body (Common Femoral vein, groin AFV/AVG). Diagnostic catheters are advanced to the cranial and caudal venous stumps and simultaneous venograms, in multiple views, are performed to define the CVO anatomy (occlusion length, diameter of the stumps, ideal location at the venous stump to start using the sharp recanalization tool, and the presence of collateral veins). To guide the SRT tools, a marker (catheter, guidewire, snare, or balloon) is typically advanced to the caudal venous stump of the CVO (cranial-to-caudal approach). However, the back end of a stiff guidewire, the Outback device, a Transseptal needle, and the RF wire are a few examples of tools that can be used in a cranial-to-caudal or a caudal-to-cranial approach. For safety reasons, and regardless of the SRT used, the operator must confirm the alignment between the SRT device and the marker tool. Typically, fluoroscopic images in antero-posterior (AP), left-anterior oblique (LAO) and right-anterior oblique (RAO) are obtained just before using the SRT tool. Alternatively, a cone beam computed tomography (CBCT) 3-D and multiplanar reconstructions, obtained with or without iodine injections at the venous stumps, are used to check for device alignment. Once the lesion is crossed successfully, a 0.035 in. stiff guidewire is placed through-and-through to facilitate subsequent balloon angioplasty (BA). In many cases, the blood vessel architecture is no longer present. Instead, there tends to be fibrotic tissue and sometimes calcifications along the CVO path, which can be refractory to BA alone due to venous recoil, to extravascular path creation and to fibrotic tissue. Therefore, primary stenting is commonly preferred in SRT cases after a 4–6 mm pre-stent BA. As there is always a risk of bleeding and it is difficult to know whether the crossed path was intravascular, extravascular, or both, covered stents are preferred especially in Superior Vena Cava (SVC) recanalizations due to risk of cardiac tamponade. Balloon expandable stents are commonly used in CVOs because they provide more precise deployment and have lower risk of stent migration in comparison with self-expandable stents which are typically reserved for malignant CVOs. The accurate deployment can prevent jailing out the contralateral brachiocephalic vein (BCV) and important chest collaterals such as the azygos vein, and inadvertent crossing of the costoclavicular junction during BCV recanalization. Historically, the venous stump diameter and occlusion length were the main factors for determining stent selection. Over time, the authors have observed that using a 10 mm in diameter stent is enough to decompress dysfunctional AVGs/AVFs and to resolve symptoms in patients with chronic and benign CVOs. The typical fibrotic tissue in CVOs prevents stents migration. Due to high venous complacency, we have observed that the baseline increased diameter of the venous stumps (12–16 mm) will reduce in size over time after CVO decompression. Therefore, intravascular ultrasound or cross sectional measurement using the baseline diameters of the dilated stumps may result in balloon/stent oversizing and increased risk of venous rupture [7].

Complications associated with SRT are similar across the different techniques. The most concerning complication is death secondary to cardiac tamponade. The pericardial recess reaches up to 4 cm superiorly from the superior cavoatrial transition, leading to a theoretical risk of tamponade during SVC recanalization. To minimize the risk of cardiac tamponade in SVC recanalization, a 4 × 40 mm angioplasty balloon is advanced over an 0.035 in. 260 cm Amplatz wire from the lower or upper venous access and positioned at the occlusion site; while, a covered stent is advanced through the other access site, over the same stiff guidewire, and is parked just before the occlusion (Table 2). Based on the reference central venogram, both margins of the stent landing zone are marked on the angiography suite monitor. Road mapping can also be utilized for guidance. Immediately after the pre-stenting BA is performed, the balloon is deflated and pulled back. Simultaneously, the covered stent is advanced to the previously defined landing zone and immediately deployed. This technical detail is believed to minimize mediastinal hemorrhage and cardiac tamponade [8]. During the recanalization of the right BCV, attention to the expansion of the lung during the respiratory cycle is important. If the lung is in the trajectory of the SRT tool, it is essential that the anesthesiologist performs an expiratory breath-hold for a few seconds while crossing with the SRT tool to ensure that the lungs and pleura are out of the way. If the lung is traversed, a pneumothorax and/or hemothorax can occur. When crossing the typically sinuous left BCV occlusions, there is a theoretical risk of injuring the supra-aortic vessels. Reviewing the baseline contrast enhanced chest computed tomography (CT) and procedural CBCT images, and/or using image fusion may minimize the risk of complications [9]. Other complications include arterial perforation with possible AVF creation, dissection and bleeding. SRT is categorized as high risk of bleeding, therefore the screening for coagulation status includes Prothrombin time/International normalized ratio (INR), Platelet count and Hemoglobin level. The thresholds are INR within < 1.5–1.8 and Platelets > 50,000/mcL blood. The authors typically perform SRT procedures under general anesthesia and in a hospital setting.

**Table 2:** Individual SRT publications—target vessel(s) details, and success and complications rates

References	SRT tool	Number of SRT procedures	Target vessel	Occlusion length	Success rate	Complication rate	Type of major complication
Liu et al. [12]	Microwire	8	5 BCV	1.9 cm (range 1.1–3.9)	100	0	N/A
			1 BCV				
			+ SVC				
			2 SVC				
Yang et al. [14]	Guidewire	16	16 SVC	2.81 ± 1.55 cm	87.50%	3 major	2 SVC injury, 1 ventricular fibrillation with death
Siddiqui et al. [17]	Chiba needle	7	SCV, BCV, SVC	N/A	85.70%	3 major	Hemothorax
							Hemopericardium
							Hemopneumothorax
Huang et al. [19]	Chiba Needle	1	1 SCV + BCV + IJ	N/A	100%	0	N/A
Zhao et al. [18]	Chiba Needle	16	16 BCV	4.4 cm (range 1–8 cm)	100%	2 minor	N/A
Cohen et al. [16]	Chiba Needle	39	3 SC	4 cm (range 1–10 cm)	95%	2 major	2 hemopericardium
			3 SC + BC			4 minor	
			2 IJ + BC				
			20 BC				
			1 BC + SVC				
			8 SVC				
Gallo et al. [6]	Chiba Needle with coaxial system	36	2 SCV	3 cm (range 0.3–5.3 cm)	94%	1 major	1 hemothorax
			3 SCV + BCV				
			15 BCV				
			10 BCV + SVC				
			6 SVC				
Wu et al. [20]	RUPS-100	14	14 SVC	0.8 ± 0.1 cm	100%	2 major	1 pericardial tamponade
						1 moderate	1 hemothorax
						1 minor	
Goo et al. [21]	RUPS-100	33	33 BCV	1.73 ± 0.8 cm (range, 1–4 cm)	93.90%	1 minor	N/A
Sun et al. [24]	Blunt impingement technique	26 Long Sheath	30	3.1 ± 1.5 cm	69.20%	2 minor	N/A
					75%		
		4 RUPS-100 Sheath					
	Guide wire	3	3		100%		
	Chiba Needle	3	2 BCV + SVC		100%		
			4 SVC				
	RUPS-100	3	12 SCV		100%		
			6 SCV + BCV				
			6 BCV				
Chen et al. [10]	Guide wire	27	27	N/A	74%	2 major	1 pericardial tamponade
	Chiba Needle	3	3 bilateral SCV		100%	4 minor	1 hemothorax
	RUPS-100	18	18 unilateral		50%		
			BCV				
			6 bilateral BCV				
Arabi et al. [26]	Transseptal Needle	7	7 BCV	N/A	100%	0	N/A
McDevitt et al. [11]	Transseptal needle (87.8% of cases)	123	59	N/A	91.50%	3 major	1 pericardial tamponade
			43 including SCV			1 moderate	1 hemothorax
			16 including SVC			7 minor	1 IVC filter occlusion
Yin et al. [25]	Transseptal needle	16	2 BCV + IJ	3 cm (range 2.3–3.6 cm)	81.30%	0	N/A
			4 BCV + SC				
			1 BCV + SVC				
Shen et al.	Transseptal needle	9	9 SVC	N/A	77%	1 major	1 pericardial tamponade
Anil et al. [27]	Outback	1	1 BCV	N/A	100%	0	N/A
Brountzos et al. [28]	Outback	2	2 SCV	N/A	100%	0	N/A
Kwon et al. [29]	Outback	11	1 SCV	N/A	90.90%	0	N/A
			9 SC + BCV				
			1 BCV				
Guimaraes et al. [8]	RF Wire	43	2 SCV	N/A	100%	1 major	1 pericardial tamponade
			29 BCV				
			8 SVC				
Iafrati et al. [33]	RF Wire	3	1 bilateral SCV + BCV	8.2 ± 3.6 cm	100%	0	N/A
			2 BCV				
Keller et al. [31]	RF Wire	20	11	4.9 cm (range 0.8–31.7)	80%	1 major	SVC laceration
			5 BCV				
			5 BCV+ SVC				
			1 SCV + BCV + SVC				
Baerlocher et al. [30]	RF Wire	1	SC	N/A	100%	0	N/A
Sivananthan et al. [32]	RF Wire	11	1 IJ	3.0 ± 2.9 cm	81%	1 major	Tracheal perforation with death
			8 BC			4 minor	
			2 SVC				
Rambhia et al. [34]	Excimer laser	1	1 BC	N/A	100%	0	N/A

*N/A* Not applicable, *SCV* subclavian vein, *BCV *brachiocephalic vein, *SVC* superior vena cava, *IJ* internal jugular vein, *RF* radio-frequency

## Back End of Stiff Guidewires

### Technical Description

**Fig. 1:**
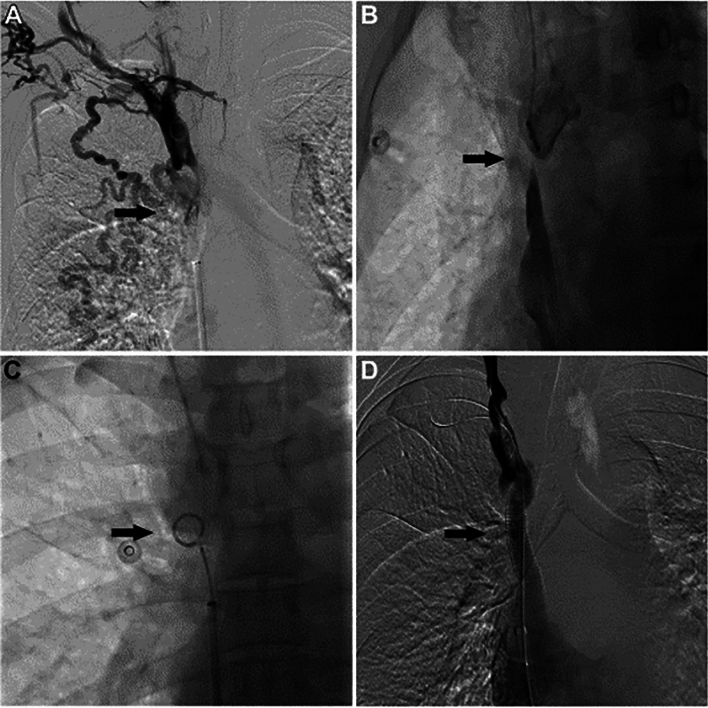
SVC recanalization using the stiff end of a guidewire. **A** SVC venogram demonstrates complete occlusion associated with venous collaterals (arrow); **B**, **C** Multiple fluoroscopic projections are used to check appropriate alignment of the guide wire and the target pigtail catheter (arrow) when crossing the occlusion from a caudal–cranial approach; **C** Note when the guide wire already had crossed the occlusion and it is overlaying the pigtail catheter; **D** Final SVC venogram shows successful stenting and recanalization of the SVC, without filling of venous collaterals [12]

The SRT using the back end of a stiff guidewire typically involves using a 0.035 in. or 0.018 in. guidewire through a 4/5-Fr (French) diagnostic catheter, and it is a cost-effective alternative. The most frequently 0.035 in. wires used are the Glidewire Hydrophilic Coated Guidewire (Terumo Medical Corp, Somerset, NJ/USA) and the Amplatz Super Stiff (Boston Scientific, Marlborough, MA/USA). The Hi-Torque Command (Abbott, Santa Clara, CA/USA) and the V18 (Boston Scientific, Marlborough, MA/USA) have been the most used 0.018 in. wires. Once the diagnostic catheter is advanced to the venous stump using conventional angiographic technique, the stiff end of the wire is carefully advanced as close as possible from either cranial-to-caudal or caudal-to-cranial approach (Fig. 1). At the opposite venous stump of the occlusion, various types of markers can be used for guidance per user preference [10]. The end of the guidewire is slowly advanced across the lesion to meet the marker, ensuring proper trajectory using fluoroscopic guidance. Once the lesion is crossed, the catheter is advanced over the wire, and intravascular position is confirmed. In very fibrotic and difficult to cross CVOs, a long introducer sheath may be required to provide adequate support.

### Best Indication

This SRT is ideally indicated for CVOs with short and a relatively straight trajectory between the venous stumps [11]. The advantages of this technique are: can be performed through multiple access points, at a relatively low cost, and the wires are available in different lengths in most interventional suites. The main disadvantage of this technique is that the stiff end typically does not navigate well inside the diagnostic catheter in tortuous venous anatomy because it has the tendency to simply move in a straight trajectory. This can be a limiting factor as CVOs frequently have a sinuous track between the venous stumps. Another disadvantage is the fact that it may require substantial force to traverse chronic and fibrotic venous occlusion, which may result in complications secondary to the perforation of CVO adjacent structures after inadvertent advancement of the stiff end beyond the target.

### Results/Complications

Liu et al. have reported the use of a 0.018 in. guidewire coaxially with a Progreat microcatheter (Terumo Medical, Tokyo/Japan) or a CXI support catheter (Cook Medical, Bloomington, IN/USA) [12]. The success rates have been reported to be 74.1–100% and the major complication rates 0–5.1% [12, 13]. Liu et al. used 0.018 in. microwires to cross CVOs with a length of 10.5–38.6 mm and reported a 100% success rate without complications in 8 patients [12]. Yang et al. treated 16 patients and had a technical success rate of 87.5%, with CVOs mean length of 2.81 ± 1.55 cm. There were 3 major complications: 2 had cardiac tamponade and 1 had transient ventricular fibrillation [14].

## Needle Assisted SRTs

### Chiba Needle

#### Technical

**Fig. 2:**
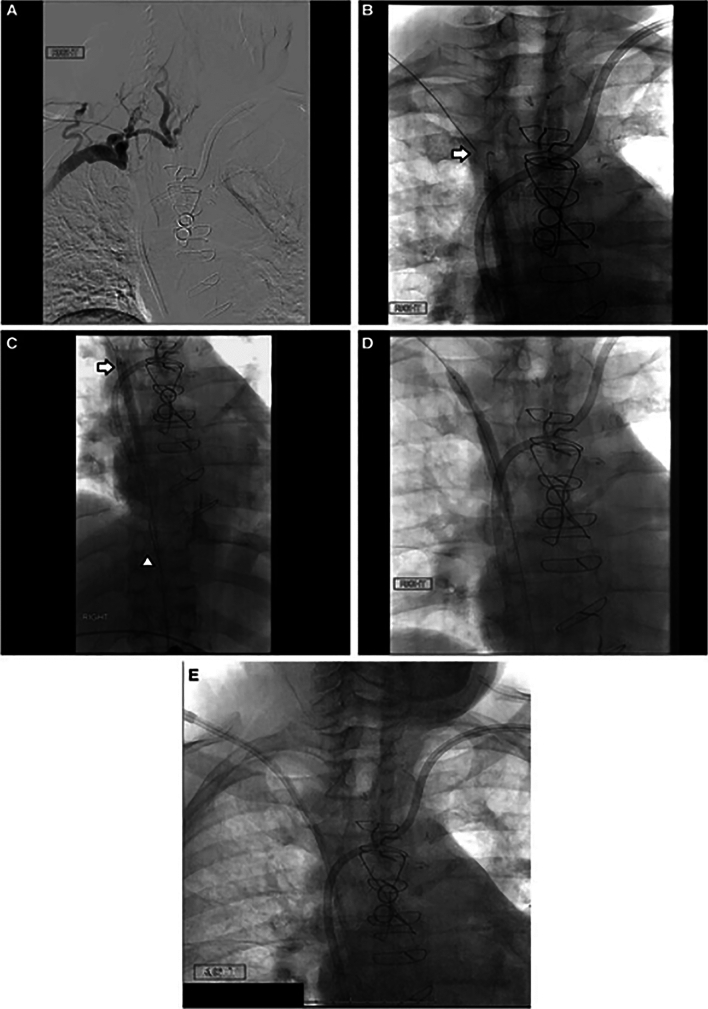
SVC recanalization with Chiba needle. **A** Central venogram through the external jugular vein demonstrates SVC occlusion with collaterals; **B** The Chiba needle (arrow) is advanced toward the target balloon under fluoroscopic guidance; **C** The Chiba needle (arrow) reaches the target patent vessel past the occlusion, and an 0.018 in. wire is passed into the IVC (arrowhead); **D** Tract dilation using balloon angioplasty; **E** Placement of a tunneled central venous catheter at the conclusion of the case [16]

The Chiba needle typically used for SRT is a hollow 21–22 G, 15–20 cm in length, stainless steel beveled needle (Cook Medical, Bloomington, IN/USA), and is another common cost-effective method. To perform this procedure, a 4-Fr sheath and 0.035 in. guidewire are advanced to the venous stump from the internal jugular or subclavian veins for a cranial–caudal approach (Fig. 2). Subsequently, from the transfemoral approach, a marker device is advanced to the caudal venous stump. The Chiba needle is then inserted into the sheath and is used to traverse the occlusion, ensuring proper trajectory under fluoroscopic guidance. Ideally, a snare or an inflated balloon can be used as a target, with puncturing of the balloon signifying intraluminal position of the needle tip [15]. Then, a stiff 0.018 in. guidewire is passed through the needle and the Chiba is replaced by a 4-Fr catheter.

### Best Indication

The ideal indication of this technique is to cross short venous occlusions with a relatively straight trajectory between the 2 stumps. The advantages of this SRT are the relatively low cost and availability in most interventional suites. The disadvantages are that it cannot be performed from upper arm or groin access because of the maximum needle length of 20 cm. Therefore, IJ and subclavian veins are the ideal accesses. Tortuous venous anatomy can be challenging as there is limited steerability of the Chiba needle tip. Similar to the technique that uses the back end of the stiff wire, the use of Chiba needle may require quite a bit of force to traverse chronic fibrotic venous occlusions. Excessive force may result in traversing more tissue than anticipated, resulting in complications [13].

### Results/Complications

Technical success rate has been 85.7–100%, with a major complication rate of 5.1–7.1% [10, 16, 17]. Cohen et al. have crossed CVOs of 33–110 mm in length and had 95% of technical success and 5% major complication rate [16]. Zhao et al. had 100% success in crossing 16 CVOs ranging 1–8 cm (4.4 cm average) and without major complications. [18]. Huang et al. reported a single case of long CVO (from the right IJ to SVC) successfully recanalized with a Chiba needle without major complications [19].

## Colapinto Needle and TIPS Set

### Technical Description

**Fig. 3:**
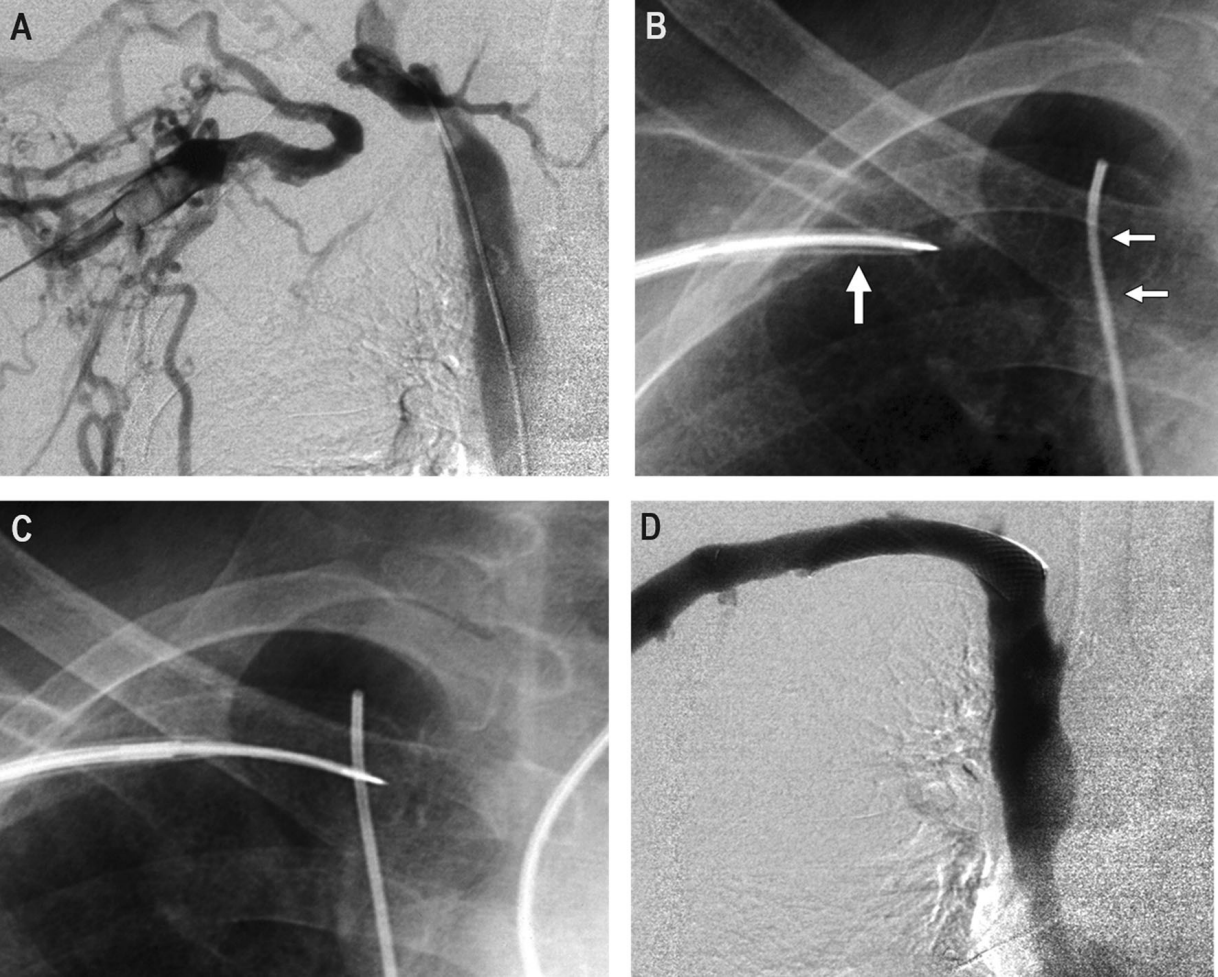
RUPS-100 recanalization of a right subclavian vein occlusion. **A** Simultaneous venograms from the right axillary vein and right BCV demonstrate a complete occlusion of the right subclavian vein-BCV transition; **B** Note a 9 Fr Rösch-Uchida (RUPS-100) curved sheath and trocar stylet in the right subclavian vein (single arrow) and a 5 Fr diagnostic catheter is placed in the right BCV as a marker (double arrows); **C** After checking the alignment between them in multiple orthogonal views, the RUPS-100 needle was advanced toward the right BCV catheter **D** Final venogram following angioplasty and stenting of the right subclavian vein [21]

The Colapinto Needle is a hollow 16 gauge (G), 50.5 cm long, stainless steel stiffening cannula with a beveled tip, and it is matched with either a 9 Fr or 10 Fr Teflon catheter. The hand-held portion of the needle is shaped with an arrow on one side, which corresponds to the angle of the needle. Therefore, the direction of needle advancement can be controlled by maneuvering the handle. The Colapinto needle is relatively stiff and therefore typically requires a right IJ approach. A TIPS set (Rösch-Uchida Transjugular Liver Access Set (RUPS-100) (Cook Medical, Bjaeverskov/Denmark) [20] can also be used as an alternative to the Colapinto needle. This device is typically used for puncture in (TIPS) procedures. The TIPS set consists of a 9 or 10 Fr introducer sheath, a trocar stylet, 20 G flexible puncture needle, and a 5-Fr catheter [21]. The Colapinto and TIPS set needles are used with a similar technique. First, the sheath is inserted either into the IJ vein or ipsilateral upper arm fistula, and the tip is advanced to the cranial venous stump over a stiff 0.035 in. guide wire (Fig. 3). Next, a trocar stylet and 5-Fr catheter are advanced to the lesion through the introducer sheath. Through a transfemoral access, a marker is advanced to the caudal venous stump and the tip of the stylet is slowly advanced across the occlusion, toward the marker, until there is no longer resistance. Once the occlusion is crossed, the trocar stylet is removed, and venography is performed for intraluminal confirmation through the 5 Fr catheter.

### Best Indication

The ideal indication is in short, straight occlusion that can be recanalized using an internal jugular or brachial vein approach. The advantage is that less force is needed to traverse the occlusion using a Colapinto needle or TIPS set in comparison with a Chiba needle, but the necessary force to cross a fibrotic venous occlusion is unknown until the tool has traversed the initial resistance. The disadvantages of using these tools for recanalization revolve around the size of the puncture in case a false passage into the mediastinum is created, and the curvature of the device tip. Additionally, the process of manually curving the set to accommodate a particular occlusion anatomy can result in kinking, which renders the set unsuitable for further use and can increase procedural expenses. If the device is to be curved, it is very difficult to recanalize from a transfemoral approach, as the sharp component requires a malleable protective component to prevent venous puncture when heading toward the occlusion [22].

### Results/Complications

Technical success rates have been reported to be 93.9–100% and major complication rate of 14.3%, primarily due to pericardial tamponade and hemothorax [20, 21, 23]. Sun et al. reported the use of Blunt impingement technique and a RUPS-100 biopsy set in 30 patients who had 100% successful recanalization rate, in CVOs measuring 3.1 ± 1.5 cm in length and had only 2 minor complications [24].

## Transseptal Needle

### Technical Description

**Fig. 4:**
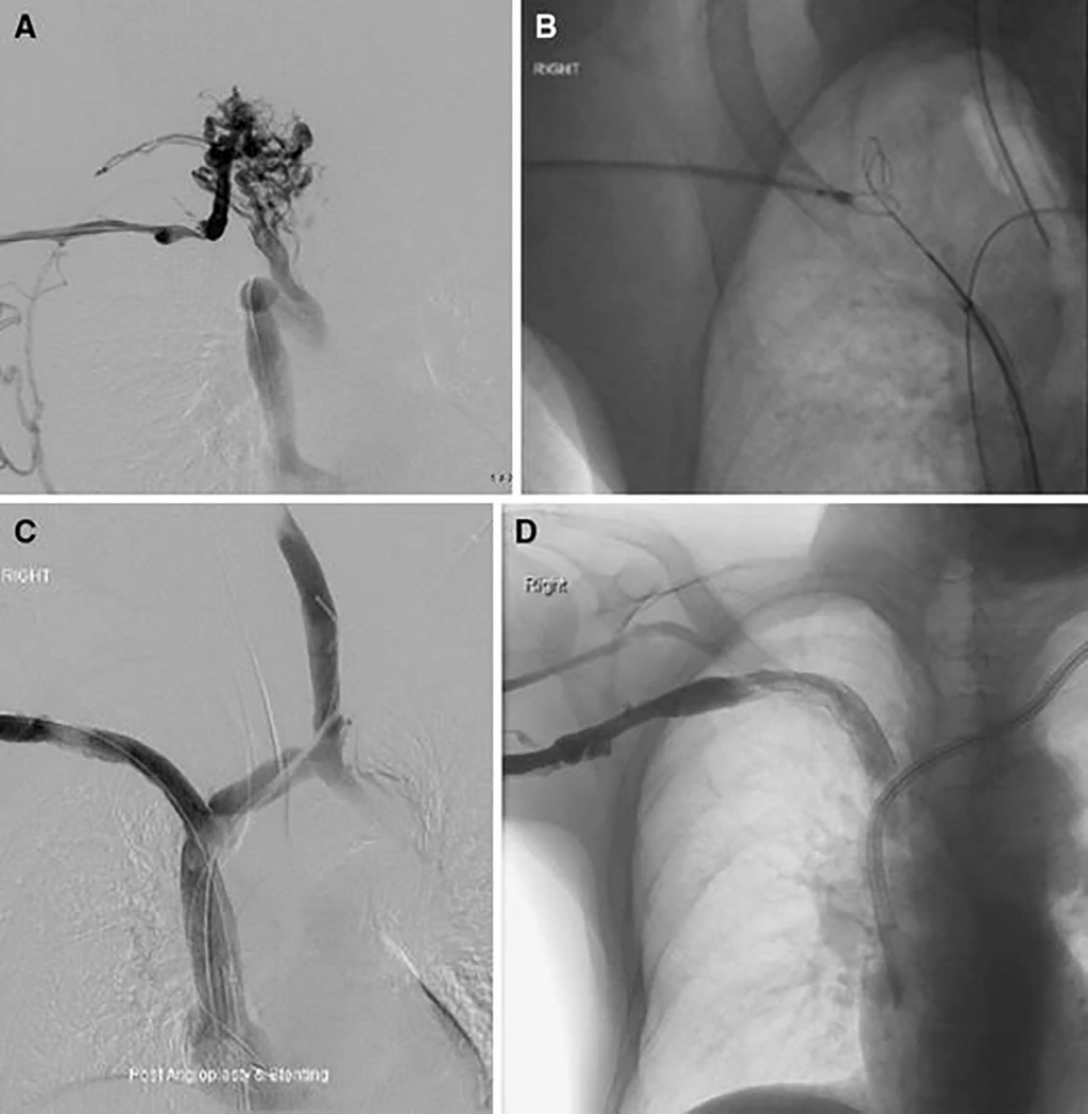
Central venous recanalization using a transseptal needle. **A** Simultaneous central venogram shows occlusion of the BCV; **B** A snare is placed in the right BCV-subclavian transition to serve as a marker for the transseptal needle using a caudal–cranial approach. A 0.018 in. wire was passed through the needle and snared out for through-and-through access; **C** Final venogram after right BCV stenting demonstrates patent central veins without collateralization; **D** One month follow up right upper extremity venography demonstrating continued patency of the right BCV stent [26]

The Transseptal needle (BRK, Abbott, Plymouth, MN/USA) is an 18 G needle, available in 71, 89 and 98 cm in length. It accepts 0.018 in. guidewires and comes with a special pre-curved 8.5-Fr sheath. It has a steerable 45° curve at the distal tip with a flexible shaft allowing adjustment according to the projected course through the mediastinum. It can be used either from a cranial-to-caudal or from a caudal-to-cranial approach. This SRT requires the use of a 8.5 Fr long introducer sheath to provide support and help with Transseptal needle orientation (Fig. 4).

### Best Indication

The ideal indication is when venous access is only available through the patient’s arm or groin. The advantages of the transseptal needle over other needles is that it has adjustable curvature with a smaller 21 G diameter needle, potentially leading to less damage in case of perforation. It has also been shown to be safe to use in occlusions that are longer than 2 cm if there is a straight trajectory, such as in most right brachiocephalic veins. It can also be used from a transfemoral or upper body approach. The disadvantage is that it can be more difficult to puncture very fibrotic, chronic occlusion with a Transseptal needle, and again, the unpredictable force that needs to be applied to the needle shaft may be excessive, which may result in inadvertent adjacent tissue puncture and injury [25].

### Results/Complications

Arabi et al. reports 7 cases which had a success rate of 100%. The major complication rate was 28.5% (2/7), consisting of one hemothorax and one cardiac tamponade, both managed successfully [26]. Yin et al. reported a technical success rate of 81.25% in 16 patients with right brachiocephalic occlusion, without major complications [26]. Shen et al. used the Transseptal needle in 9 CVOs and had 77.7% of success rate and 1 cardiac tamponade [25]. McDevitt et al. published the largest experience with Transseptal needle in the recanalization of CVOs. Transseptal needle was used in 108/123 (87.8%) and had a global technical success of 90.2%. Severe complications rate was 2.4%: one pericardial tamponade and one hemothorax [11].

## Coaxial Needle Systems

### Technical Description

**Fig. 5:**
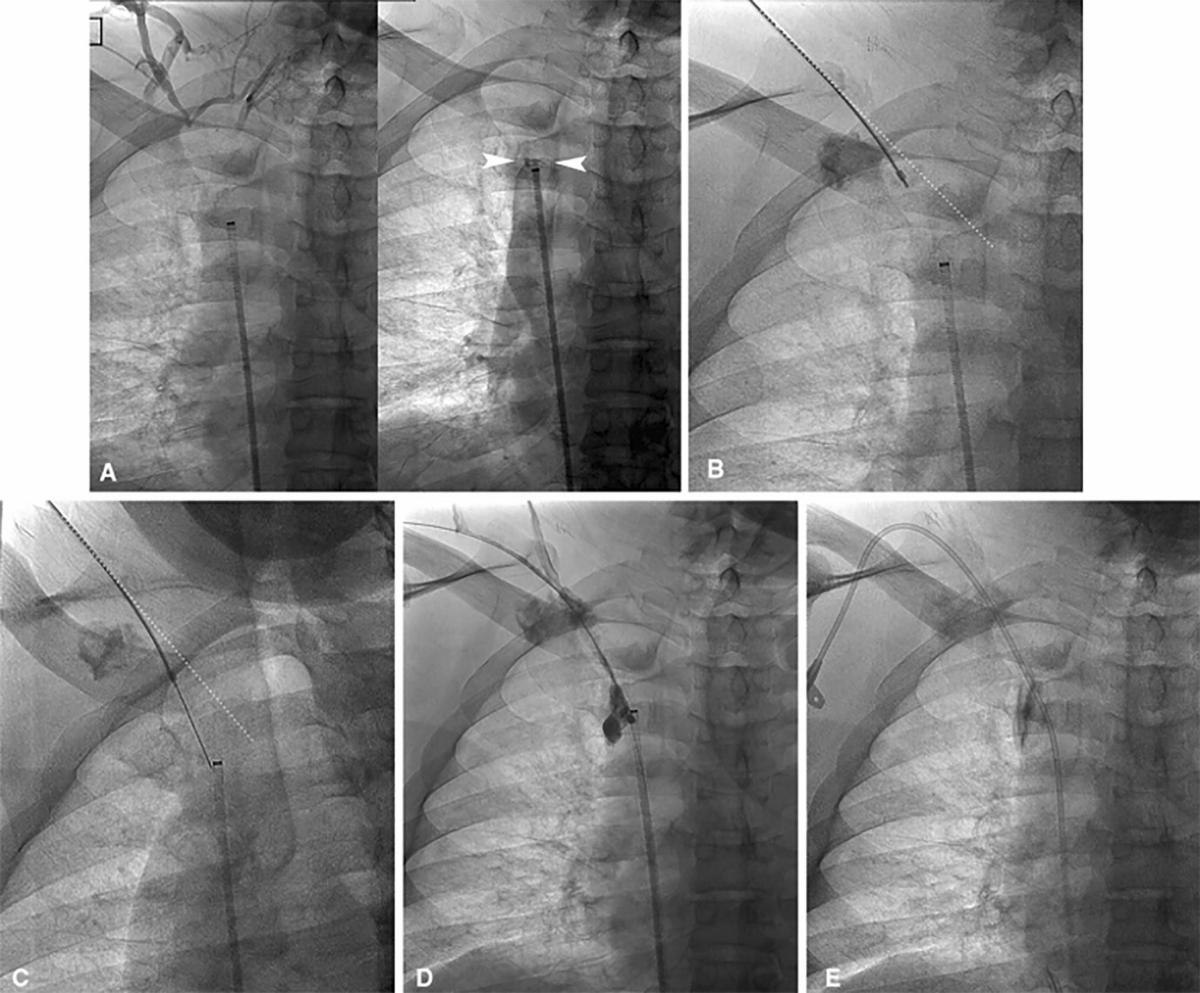
Chiba needle through a coaxial system right BCV recanalization. **A** Initial central venograms from the right external jugular vein (EJV) and from the cranial aspect of the SVC demonstrates an occluded right BCV (white arrowheads); **B** Through the EJV, an 18 G trocar needle was manually curved to optimize trajectory toward sheath positioned in the SVC (marker). The dotted line demonstrates the path the Chiba needle would have taken without use of the coaxial system; **C** After checking alignment using multiple orthogonal views, a 22 G Chiba needle is inserted through the trocar, traversing the occlusion to meet the target; **D** Venogram after crossing demonstrates contrast pooling in the CVO, without extravasation into adjacent vital structures; **E** Note a tunneled central venous catheter was placed in the right EJV for later transition to a surgically placed HeRO graft [6]

The coaxial system uses a modified Chiba needle technique that provides a more directional control from a supraclavicular approach [6]. A 15 cm, 18 G trocar needle (Cook Medical; Bloomington, IN/USA) is curved to the orientation and length of the occlusion (Fig. 5). A 5 Fr sheath is inserted into the IJ vein, the inner trocar stylet is removed, and using a guidewire the trocar needle is advanced to the level of the occlusion. A marker is advanced to the caudal venous stump using a transfemoral approach to serve as a target. Once confirming proper trajectory in multiple fluoroscopic orthogonal views, a 20 or 25 cm, 21 or 22 G Chiba needle (Cook Medical, Bloomington, IN/USA) is then inserted coaxially through the trocar needle, across the occlusion, and toward the target. A 0.014 or 0.018 in. guidewire is passed through the Chiba needle to the target. A 4 (Fr) Accustick triaxial introducer system (Boston Scientific, Natick, MA/USA) is advanced across the occlusion.

### Best Indication

The ideal indication to perform this technique safely is in the setting of a straight and short right brachiocephalic venous occlusion. The main advantages of the coaxial needle system are the fact that it is relatively cheaper (in comparison with an Outback device, for example), and that a 21 or 22 G Chiba needle allows the advancement of a 0.018 in. guide wire instead of a 0.035 in. system. The ability to curve the trocar needle allows more directional control of the Chiba needle [6]. The disadvantage of the coaxial needle assisted SRT is that it requires a relatively straight trajectory (IJ access is preferred instead of upper arm venous access or subclavian access), and that the recanalization cannot be performed from the groin or arm access because of limited needle length. Therefore, it is primarily limited to the recanalization of the right brachiocephalic vein and superior vena cava. Also, some CVOs can have chronic, well-organized fibrotic tissue, which may pose significant resistance and inadvertent puncture of adjacent structures.

### Results/Complications

Gallo et al. reported successful recanalization of lesions with a mean length of 30 mm. The success rates were as high as 94%, and major complication rate of 3%, primarily due to hemothorax [6].

## Outback Re-entry Device

### Technical Description

**Fig. 6:**
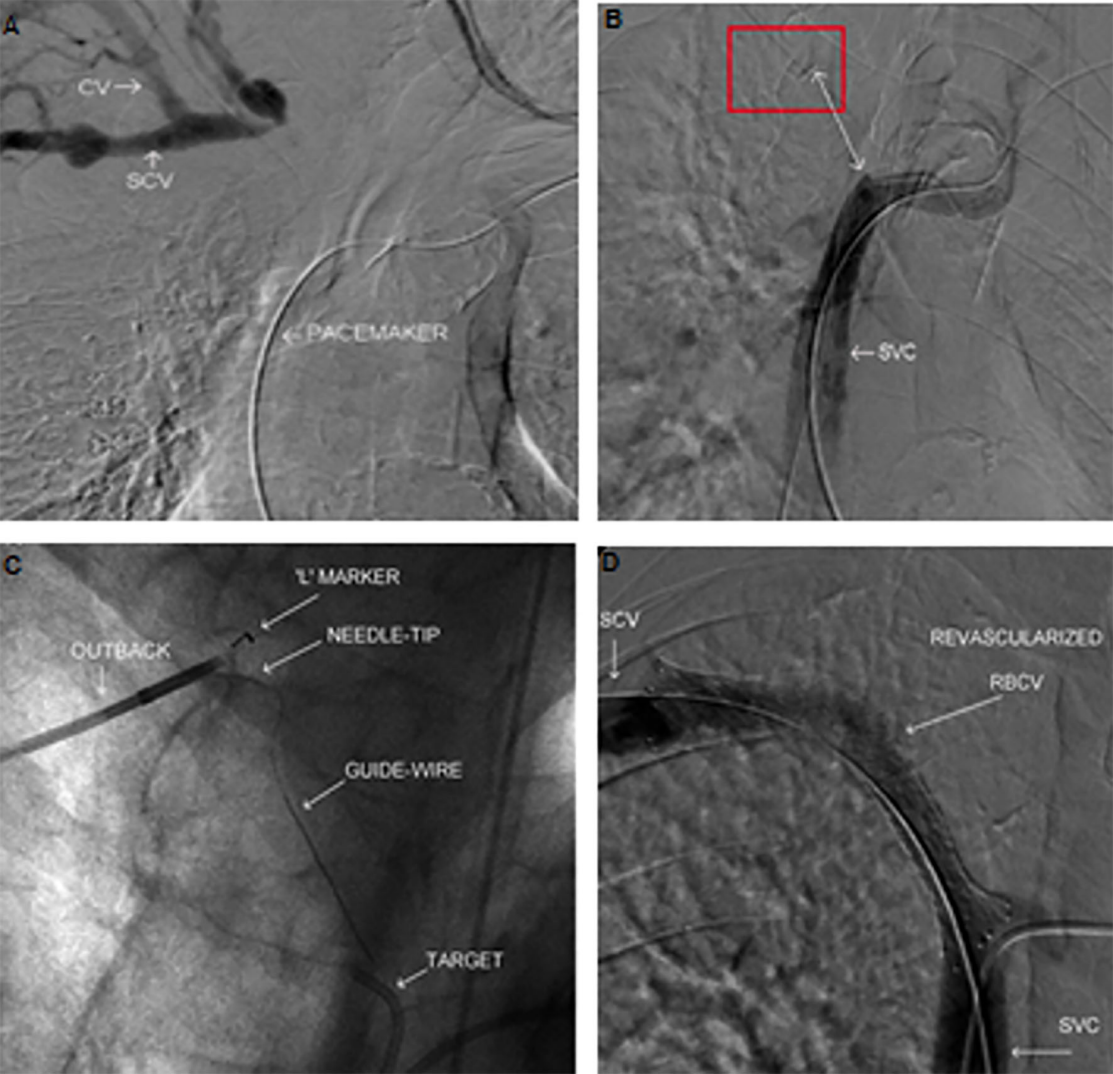
Recanalization of a right BCV using an Outback-LTD re-entry catheter. **A**, **B** Initial central venograms demonstrate right BCV occlusion. The double headed arrow designates the length of occlusion, and the red box indicates the location of the cranial venous stump. Incidentally noted pacemaker lead and the patent SVC are labeled; **C** The Outback-LTD device was advanced to the right subclavian vein and positioned so that the “L” locator marker is pointed toward the guidewire used to cross the occluded segment into the cranial venous stump. Through the lateral exit port of the Outback catheter, the curve needle tip exits with an 0.014 in. wire traveling through the recanalized track; **D** Final venogram of the recanalized right BCV after stenting, without evidence of collateral venous filling or contrast extravasation [27]

The 6 Fr Outback LTD device (Cordis, Miami Lakes, FL/USA) has been designed as a re-entry tool that allows a guidewire to get back into the true arterial lumen from the subintimal space. In the venous system, the Outback can be used either from a cranial-to-caudal or a caudal-to-cranial approach. The occluded track is typically partially recanalized with a 0.035 in. Glidewire Hydrophilic Coated Guidewire (Terumo Medical Corp, Somerset, NJ/USA) and the Outback is primarily used as the re-entry tool (Fig. 6). A marker is placed at the opposite venous stump to serve as a target. The catheter is then advanced toward the occlusion until resistance is met. With multiple angle fluoroscopic guided images, the Outback radiopaque ‘L’ marker is oriented toward the target. At this point a 22 G nitinol needle housed at the tip is advanced from the extravascular space into the opposite venous stump lumen and a 0.014 in. guidewire is passed through the venous occlusion into the lumen.

### Best Indication

The advantage of this device is that it is flexible, torquable, and has a curved needle tip allowing recanalization of curved occlusions. Additionally, recanalization can be performed from the IJ, brachial or femoral vein access. The disadvantages are: it is expensive, and that there is very limited data on the off label use of the device.

### Results/Complications

Three cases have been published with 100% success in re-establishing flow by adapting a similar technique used to regain access to the true lumen in arterial recanalization [27, 28]. Kwon et al. have published an abstract that demonstrated the Outback device use as a SRT tool in 11 patients, in which subclavian and innominate veins were recanalized [29]. The re-entry device was introduced via the outflow vein of the arm, femoral or IJ veins. The technical success rate was 90.9–100%, with no major complications [27, 28].

## Radiofrequency Wire (Sniper Technique)

### Technical Description

**Fig. 7:**
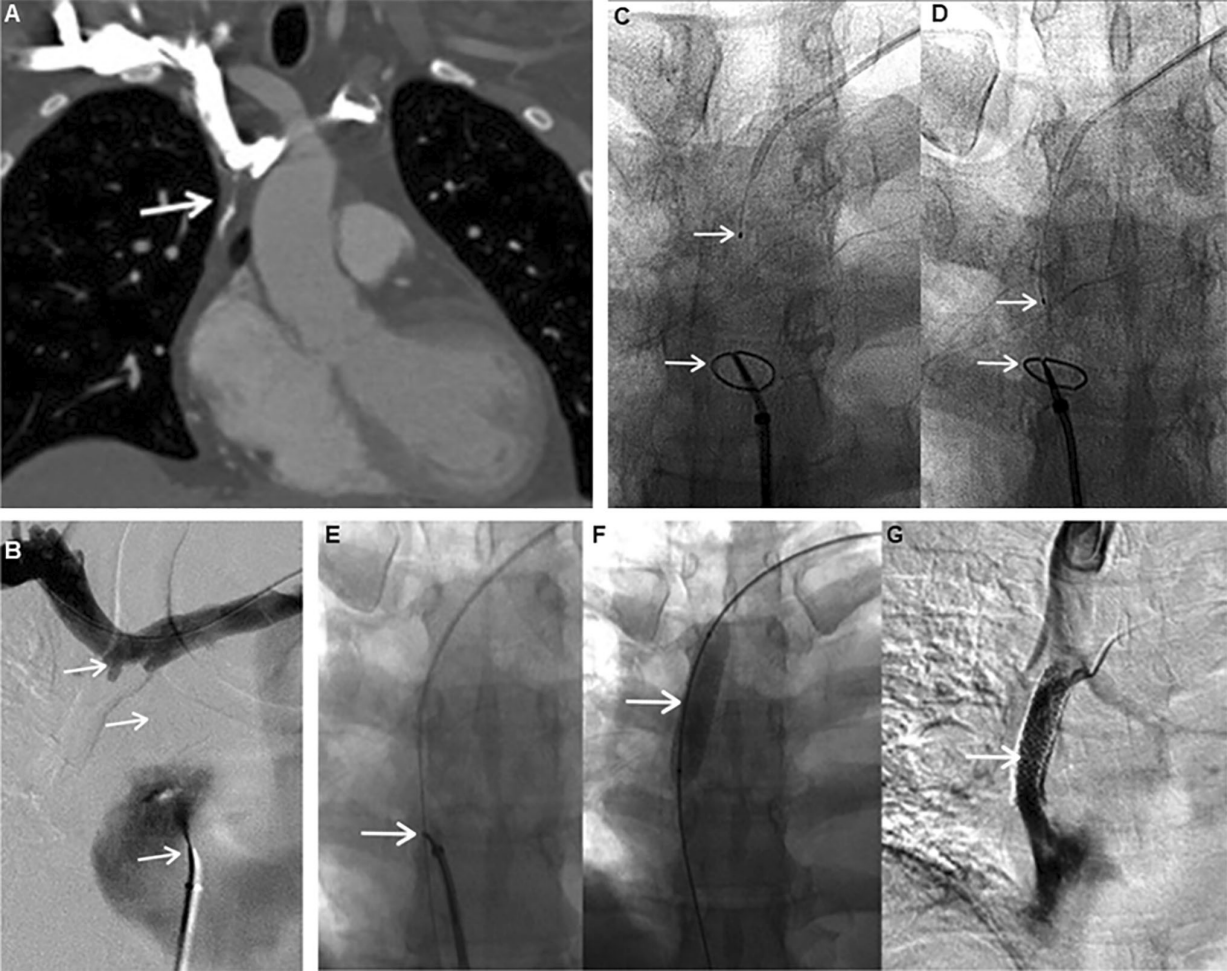
Sniper technique for recanalization of a long segment superior vena cava occlusion. **A** Coronal CTA of the chest demonstrates a long segment occlusion of the superior vena cava, which extends from the brachiocephalic confluence to the right atrium. Faint linear contrast opacification is noted extending from the brachiocephalic confluence, which was the sequela of a prior recanalization attempt (white arrow); **B** Central venogram performed simultaneously from above (top white arrow) and below (bottom arrow) the occluded SVC (middle white arrow); **C**, **D** The radiofrequency wire (upper white arrows) and the target snare (bottom arrows) in alignment with incremental advancement toward the snare, ensuring proper trajectory with multiple fluoroscopic projections; **E**–**G** Successful traversal of the radiofrequency wire through the occlusion, which was snared out (left white arrow) for through-and-through access for subsequent angioplasty (middle white arrow) and stenting (right white arrow) of the SVC, which demonstrated patent stent without extravasation on final venogram [8]

Sharp recanalization using a radiofrequency wire is referred to as the Sniper Technique (ST). The PowerWire (Baylis Medical, Mississauga, ON/Canada) is 0.035 in. 250 cm guidewire that delivers radiofrequency energy at the tip, which can be straight or curved (angled tip of 20, 30 and 40°). The monopolar generator is attached to the guidewire through a cable and a grounding pad which closes the energy circuit. The typical ST begins by obtaining venous access through a patent AVF/AVG or through the brachial, basilic or IJ veins ipsilateral to the CVO and a second venous access via femoral approach. A 40 cm (IJ access)/65 cm (upper arm access) or 100 cm (from femoral access) 5 Fr KMP (Cook Medical, Bloomington, IN/USA) diagnostic catheter is used to obtain access to the cranial and caudal venous stumps, respectively. In most cases, a 65 cm long introducer sheath is used to provide stable femoral access (Pinnacle Destination, Terumo Medical Corporation, Somerset/NJ—USA). While a straight PowerWire is advanced to one of the KMP catheters tip, a 10 mm in diameter, 120 cm long snare (Amplatz Goose Neck™ snare, Medtronic, Minneapolis, MN/USA) is exposed immediately outside of the other KMP catheter positioned at the opposite venous stump and used as a target. Simultaneous contrast hand injections are performed for central venograms using CBCT or during multiple orthogonal views so that the correct venous stumps are confirmed, and to decide if a cranial–caudal or caudal–cranial approach would be more appropriate (Fig. 7). Once the alignment between the PowerWire and the snare has been verified with fluoroscopy in AP, RAO and LAO projection or by CBCT, the PowerWire is slowly advanced a few millimeters each time (2 s active, continuous mode), with a trajectory checked intermittently in multiple views. The PowerWire can cross any venous occlusion secondary to RF energy delivery at the tip of the wire, which creates a 0.035 in. channel toward the snare. Once the occlusion is crossed, the PowerWire is snared out for through-and-through access and is subsequently exchanged for a 0.035 in stiff wire for BA and stenting [8].

### Best Indication

The ideal indication is for short, chronic, right or left brachiocephalic vein and SVC occlusions, and the authors have used the ST successfully in CVOs up to 10 cm in length. The ST has a few advantages. Because the PowerWire has a relatively flexible tip and a length of 250 cm, it can be used through any venous access and advanced either in a cranial-to-caudal or caudal-to-cranial direction. Also, some CVOs have chronic and well-organized fibrotic tissue that may provide significant resistance that can, however, be easily (and in the authors experience safely) traversed with appropriate guidance using a curved diagnostic catheter combined with a straight PowerWire in most cases. The disadvantage of the ST is that it has limited availability in typical angiography suites, and it can be more expensive comparatively to most of the needle assisted SRTs.

### Results/Complications

Sixty-eight CVOs in 66 patients have been reported with 91% technical success rate (80–100%) [8, 30–33].

## Excimer Laser

### Technical Description

The use of the excimer laser as a SRT tool has been reported in a single left brachiocephalic venous recanalization. A 9 Fr Agilys angulated sheath (Stryker, Portage, MI/USA) is introduced coaxially through a 14 Fr straight sheath and a 9 mm angioplasty balloon is advanced up to the occlusion and then lightly inflated to center its wire port in the vessel. Through the inflated balloon’s wire port, the 0.9-mm Excimer laser fiber (Spectranetics, Colorado Springs, CO/USA) with a 0.014 in. High-Torque Spartacore wire (Abbott, Plymouth, MN/USA) are loaded into it and advanced to the occlusion. Using interrupted laser pulses, continuous rechecking, and venograms, the laser fiber and wire were slowly advanced aiming toward a 6-F sheath at the opposite venous stump. The wire was then snared for body flossing technique. After removing the 9 mm balloon, several passes through the occlusion using the laser were performed with the 0.9 mm laser fiber. A 2.5 mm laser fiber was then passed for a total of 5 times. Once a channel was created, exchanged for a stiff 0.035 in. wire is performed to provide support for BA and stenting and or for venous access catheter placement.

### Best Indication

There is limited experience with this SRT. Some of the limitations of this approach are the need for an Excimer laser, multiple sheaths, and wires. The Excimer has limited availability in most angiography suites. Also, according to the authors, this technique requires advanced endovascular skills as there is a potential risk of adjacent organ perforation [34].

### Results/Complications

One successful case has been reported in the literature.

## Summary

There are several effective SRTs to treat CVOs after failed attempt using conventional techniques. The complication rates are variable, relatively low, and it seems to depend on a few factors such as the SRT tool used, operator experience and CVO characteristics. Regardless the SRT used, meticulous technique with the preparation to manage potential life-threatening events is critical.

## References

[1] Hovsepian DM, Bonn J, Eschelman DJ. Techniques for peripherally inserted central venous catheters. J Vasc Interv Radiol. 1993;4:795–803. 8281003 10.1016/s1051-0443(93)71976-4

[2] Allen AW, Megargell JL, Brown DB, et al. Venous thrombosis associated with the placement of peripherally inserted central catheters. J Vasc Interv Radiol. 2000;11:1309–14. 11099241 10.1016/s1051-0443(07)61307-4

[3] Schwab SJ, Quarles LD, Middleton JP, Cohan RH, Saeed M, Dennis VW. Hemodialysis-associated subclavian vein stenosis. Kidney Int. 1988;33(6):1156–9. 10.1038/ki.1988.124. 2969991 10.1038/ki.1988.124

[4] Criado E, Marston WA, Jaques PF, Mauro MA, Keagy BA. Proximal venous outflow obstruction in patients with upper extremity arteriovenous dialysis access. Ann Vasc Surg. 1994;8(6):530–5. 10.1007/BF020.17408. 7865390 10.1007/BF02017408

[5] Ferral H, Bjarnason H, Wholey M, Lopera J, Maynar M, Castaneda-Zuniga WR. Recanalization of occluded veins to provide access for central catheter placement. J Vasc Interv Radiol. 1996;7(5):681–5. 8897332 10.1016/s1051-0443(96)70828-x

[6] Gallo CJR, Ronald J, Pabon-Ramos WM, Suhocki PV, Sag AA, Martin JG, Smith TP, Kim CY. Sharp recanalization of chronic central venous occlusions of the thorax using a steerable coaxial needle technique from a supraclavicular approach. Cardiovasc Intervent Radiol. 2021;44(5):784–8. 33388871 10.1007/s00270-020-02728-7

[7] Funaki B. Superior vena cava rupture and pericardial tamponade. J Vasc Interv Radiol. 2023;34(7):1283–4. 36702377 10.1016/j.jvir.2023.01.019

[8] Guimaraes M, Schonholz C, Hannegan C, Anderson MB, Shi J, Selby B Jr. Radiofrequency wire for the recanalization of central vein occlusions that have failed conventional endovascular techniques. J Vasc Interv Radiol. 2012;23(8):1016–21. 10.1016/j.jvir.2012.05.049. 22739648 10.1016/j.jvir.2012.05.049

[9] Yamada R, Bassaco B, Wise C, Barnes L, Golchin N, Guimaraes M. Radiofrequency wire technique and image fusion in the creation of an endovascular bypass to treat chronic central venous occlusion. J Vasc Surg Cases Innov Tech. 2019;5(3):356–9. 31440713 10.1016/j.jvscit.2019.06.011 PMC6699193

[10] Chen B, Lin R, Dai H, et al. Sharp recanalization for treatment of central venous occlusive disease in hemodialysis patients. J Vasc Surg Venous Lymphat Disord. 2022;10(2):306–12. 10.1016/j.jvsv.2021.08.007. 34438087 10.1016/j.jvsv.2021.08.007

[11] McDevitt JL, Srinivasa RN, Gemmete JJ, Hage AN, Srinivasa RN, Bundy JJ, et al. Approach, technical success, complications, and stent patency of sharp recanalization for the treatment of chronic venous occlusive disease: experience in 123 patients. Cardiovasc Intervent Radiol. 2019;42:205–12. 30460385 10.1007/s00270-018-2090-1

[12] Liu Z, Tang Y, Huang J, et al. Efficacy and safety of sharp recanalization with the stiff end of a microguidewire for treatment of refractory central venous occlusions in Hemodialysis patients. Ann Vasc Surg. 2024;98:398–405. 10.1016/j.avsg.2023.08.029. 37858667 10.1016/j.avsg.2023.08.029

[13] Chen B, Lin R, Dai H, et al. XperCT facilitates sharp recanalization for the treatment of chronic thoracic venous occlusive disease in hemodialysis patients. J Vasc Access. 2023. 10.1177/11297298231151459. 36708010 10.1177/11297298231151459

[14] Yang L, et al. The feasibility and safety of sharp recanalization for superior vena cava occlusion in hemodialysis patients: a retrospective cohort study. Hemodial Int. 2020;24(1):52–60. 10.1111/hdi.12804. 31808994 10.1111/hdi.12804

[15] Yoong GSW, Koh FHX, Wee BBK, et al. How to do it: value-driven sharp recanalization of central vein occlusion. ANZ J Surg. 2020;90:362–3. 31782220 10.1111/ans.15599

[16] Cohen EI, Beck C, Garcia J, et al. Success rate and complications of sharp recanalization for treatment of central venous occlusions. Cardiovasc Intervent Radiol. 2018;41(1):73–9. 10.1007/s00270-017-1787-x. 28879566 10.1007/s00270-017-1787-x

[17] Siddiqui M, Ahearn B, Joseph A, et al. Abstract No. 608 Endovascular recanalization of central venous occlusions in dialysis patients. J Vasc Interv Radiol. 2022;33(6):S228–9. 10.1016/j.jvir.2022.03.590.

[18] Zhao Y, et al. Sharp recanalization of the brachiocephalic vein occlusion through the external jugular vein in hemodialysis patients. Ann Transl Med. 2020;8(10):640. 10.21037/atm-20-3015. 32566577 10.21037/atm-20-3015 PMC7290652

[19] Huang XR, et al. Percutaneous superior vena cava puncture successful recanalization of a long-segment, angled central venous occlusion: a case report. Ann Palliat Med. 2022;11(6):2139–43. 10.21037/apm-22-529. 35817747 10.21037/apm-22-529

[20] Wu XW, Zhao XY, Li X, et al. Effectiveness of sharp recanalization of superior vena cava-right atrium junction occlusion. World J Clin Cases. 2021;9(16):3848–57. 10.12998/wjcc.v9.i16.3848. 34141741 10.12998/wjcc.v9.i16.3848 PMC8180228

[21] Goo DE, Kim YJ, Choi DL. Use of a Rosch-Uchida needle for recanalization of refractory dialysis-related central vein occlusion. AJR Am J Roentgenol. 2010;194(05):1352–6. 20410425 10.2214/AJR.09.3485

[22] Lang EV, Vrachliotis TG, Brophy DP. Sharp recanalization for chronic central venous occlusions. Tech Vasc Interv Radiol. 2000;3(1):21–8. 10.4053/tv.2000.5342.

[23] Honnef D, Wingen M, Günther RW, et al. Sharp central venous recanalization by means of a TIPS needle. Cardiovasc Intervent Radiol. 2005;28:673–6. 10.1007/s00270-004-0323-y. 16091988 10.1007/s00270-004-0323-y

[24] Sun JB, et al. The efficacy and safety of blunt impingement followed by a sharp recanalization technique in hemodialysis patients with refractory central vein occlusion: a single-center experience. Ann Transl Med. 2022;10(14):768. 10.21037/atm-22-3131. 35965835 10.21037/atm-22-3131 PMC9372654

[25] Yin X, Shen X, Zhou Z, Chen Q, Zhou L, Cui T. Efficacy and safety of recanalization with transseptal needle for chronic total occlusion of the brachiocephalic vein in hemodialysis patients. Ann Transl Med. 2020;8(18):1141. 10.21037/atm-20-5369. 33240990 10.21037/atm-20-5369 PMC7576017

[26] Arabi M, Ahmed I, Mathami A, Ahmed D, Aslam N. Sharp central venous recanalization in hemodialysis patients: a single-institution experience. Cardiovasc Intervent Radiol. 2016;39:927–34. 26676109 10.1007/s00270-015-1270-5

[27] Anil G, Taneja M. Revascularization of an occluded brachiocephalic vein using Outback-LTD re-entry catheter. J Vasc Surg. 2010;52(4):1038–40. 20598479 10.1016/j.jvs.2010.04.056

[28] Brountzos EN, Preza O, Kelekis A, Panagiotou I, Kelekis N. Recanalization of dialysis catheter-related subclavian vein occlusion using a re-entry device: report of two patients. Cardiovasc Interv Radiol. 2011;34:207–11. 10.1007/s00270-010-9955-2 20694466

[29] Kwon Y, et al. Outback LTD re-entry device for endovascular recanalization of central venous occlusions associated with failing hemodialysis access. Arab J Interv Radiol. 2020;4(3):545. 10.1055/s-0041-1729113. 10.1111/hdi.13262 40395037

[30] Baerlocher MO, Asch MR, Myers A. Successful recanalization of a longstanding complete left subclavian vein occlusion by radiofrequency perforation with use of a radiofrequency guide wire. J Vasc Interv Radiol. 2006;17(10):1703–6. 17057015 10.1097/01.RVI.0000243637.23923.A7

[31] Keller EJ, Gupta SA, Bondarev S, Sato KT, Vogelzang RL, Resnick SA. Single-center retrospective review of radiofrequency wire recanalization of refractory central venous occlusions. J Vasc Interv Radiol. 2018;29(11):1571–7. 10.1016/j.jvir.2018.06.017. 30293732 10.1016/j.jvir.2018.06.017

[32] Sivananthan G, MacArthur DH, Daly KP, Allen DW, Hakham S, Halin NJ. Safety and efficacy of radiofrequency wire recanalization of chronic central venous occlusions. J Vasc Access. 2015;16(4):309–14. 10.5301/jva.5000360. 25656250 10.5301/jva.5000360

[33] Iafrati M, Maloney S, Halin N. Radiofrequency thermal wire is a useful adjunct to treat chronic central venous occlusions. J Vasc Surg. 2012;55(2):603–6. 10.1016/j.jvs.2011.09.090. 22104339 10.1016/j.jvs.2011.09.090

[34] Rambhia S, Janko M, Hacker RI. Laser recanalization of central venous occlusion to salvage a threatened arteriovenous fistula. Ann Vasc Surg. 2018;50:297e1–3. 10.1016/j.avsg.2017.11.043. 10.1016/j.avsg.2017.11.043 29455013

